# Effects of dynamic taping and moderate-intensity TheraBand training *versus* usual care on pain, disability, and well-being in adult National Cadet Corps with chronic heel pain: a pilot and feasibility trial

**DOI:** 10.7717/peerj.20777

**Published:** 2026-03-06

**Authors:** Samsudin Choudhary, Mohammad Sidiq, Jyoti Sharma, Faizan Kashoo, Aksh Chahal, Balamurugan Janakiraman, Rajkumar Krishnan Vasanthi, Abhishek Sharma, Monira I. Aldhahi

**Affiliations:** 1 Department of Physiotherapy, School of Allied Health Sciences, Galgotias University, Greater Noida, Uttar Pradesh, India; 2 Physiotherapy Department, Tishk International University, Erbil, Iraq; 3 Department of Physical Therapy and Health Rehabilitation, College of Applied Medical Science, Majmaah University, Majmaah, Al Majma’ah, Saudi Arabia; 4 SRM College of Physiotherapy, Faculty of Medicine and Health Sciences, SRM Institute of Science and Technology (SRMIST), Kattankulathur, Tamil Nadu, India; 5 Faculty of Health and Life Sciences, INTI International University, Nilai, Negeri Sembilan, Malaysia; 6 Arogyam Institute of Paramedical and Allied Sciences, Arogyam Medical College and Hospital, Haridwar, Uttarakhand, India; 7 Department of Rehabilitation Sciences, College of Health and Rehabilitation Sciences, Princess Nourah bint Abdulrahman University, Riyadh, Saudi Arabia

**Keywords:** Heel pain, Plantar fasciitis, Elastic therapeutic tape, Resistance training, Exercise therapy, Physical therapy modalities, Quality of life, Pain measurement, Pilot projects, Young adult

## Abstract

**Background:**

Chronic heel pain (CHP), often attributed to plantar fasciitis, is prevalent among physically active individuals such as National Cadet Corps (NCC) cadets. While Dynamic Taping (DT) and TheraBand exercises have each demonstrated individual benefits in musculoskeletal rehabilitation, their combined effect remains underexplored. This pilot study aimed to evaluate the feasibility and therapeutic effects of combining Dynamic Taping with moderate-intensity TheraBand training on pain, disability, and overall well-being among NCC cadets with chronic heel pain.

**Methods:**

This feasibility trial was conducted at Galgotias University, which granted ethical approval with reference number DRC/PT/UG/24-25/011, and it was prospectively registered with Clinical Trial Registry India (CTRI//2024/10/074693). Twenty NCC cadets (mean age 20.2 years) with ≥3 months of heel pain participated in a 4-week intervention. Outcomes included the Foot Function Index (FFI) and the Short Form-36 Health Survey (SF-36), assessed pre- and post-intervention. Paired t-tests were used for statistical analysis.

**Results:**

Multivariate analysis of covariance (MANCOVA) controlling for baseline scores revealed no significant between-group differences in Foot Function Index or SF-36 outcomes (Pillai’s Trace = 0.750, *p* = .103). Univariate analyses confirmed non-significant group effects for all individual measures after Bonferroni correction (all *p* > .008), indicating the intervention did not produce statistically superior outcomes compared to the control condition. Feasibility criteria were met with 83% recruitment success, 100% participant retention, intervention adherence of 89.2%–91.5%, and 95% questionnaire completion rates. No adverse events were reported.

**Conclusion:**

The integration of dynamic taping with moderate-intensity TheraBand exercises seem a feasible and promising approach in managing chronic heel pain among active young adults. These results underscore the need for larger, controlled trials to confirm the findings and examine long-term effects.

## Introduction

Chronic heel pain (CHP) is a multifaceted ailment frequently linked to plantar fasciitis, though other causes such as nerve entrapment, infection, or rarely, lymphoma may also be implicated (Ata & Altunay, 2024; Safarpour, Mittal & Jabbari, 2023; Coles et al., 2021; Horner et al., 2022; Effendi et al., 2016). Musculoskeletal (MSK) disorders such as plantar fasciitis (PF) and Achilles tendinopathy (AT) represent leading causes of disability globally, as documented in the Global Burden of Disease (GBD) study (Gill et al., 2023; Liu et al., 2025). The effects of MSK conditions on overall quality of life are immense, with ruinous effect not only on physical capabilities especially in those who are athletes, obese and also those who work on prolonged occupational activities (Hirschmüller & Weidermann, 2022; Agyekum & Ma, 2015). The conditions are widespread around the world, and they lead to severe disability and long-term pain, as well as they are the primary cause of physical exhaustion.

Musculoskeletal treatment is complex, including both conservative and surgical intervention, with large socioeconomic repercussions (Kim & Lee, 2023; Bourke et al., 2024; Latt et al., 2020). Lower leg and ankle injury is particularly common in young active people like Indian National Cadet Corps (NCC) cadets due to frequent repetitive mechanical stress and high physical activity demands (Waterman, Dunn & Orr, 2016; Kuprinenko et al., 2022). Rehabilitation of lower limb injuries in athletes, military individuals and NCC cadets is an essential measure in recovery and prevention of subsequent injuries (Sergeev & Piskareva, 2024). Such protocols are personalized according to the individual needs in terms of what kind of injuries someone has and what kind of activities that someone requires (Zhao & Wang, 2023). The study reports the significance of a personalized approach to rehabilitation, which combines physical and medical solutions to maximize the recovery results. Such practice is vital to athletes and NCC cadets, as the latter is also exposed to a high risk of injuries to lower limbs associated with the physically challenging nature of their jobs (Kuprinenko & Tymruk-Skoropad, 2023; Waterman, Dunn & Orr, 2016). These practices highlight the relevance of grasping pathophysiology of frequent injuries and integrating intervention countermeasures to reduce risks and improve recovery (Michelsson et al., 2005).

Dynamic taping (DT) and moderate intensity TheraBand training (TBT) are often used adjunctive measures in the management of chronic heel pain (Yoo, 2016). DT is a type of elastic cotton tape which features high recoil properties and is intended to replicate the action of muscles, thus minimizing mechanical overload and maximizing proprioceptive feedback without any restriction of movement (Chang et al., 2025). The conjectured physiological mechanism is redistribution of load across the kinetic chain, decrease in tissue strain and optimization of movement patterns (Szeles & Green, 2025). TBT on the other hand offers progressive resistance training which encourages eccentric strengthening, enhances flexibility and neuromuscular control of the lower limb musculature (Anwer et al., 2021; Apaydın & Kırmızı, 2024; Pawik et al., 2022).

Studies have shown that both these approaches can help decrease pain and increase function in such conditions as plantar fasciitis and Achilles tendinopathy (Kim & Lee, 2023; Castro-Méndez et al., 2022). The combined efficacy of DT and moderate-intensity TBT in the management of foot and ankle injuries is an emerging field, with scant literature available especially in the Indian healthcare perspective. DT With its biomechanical properties, provides multidirectional joint support and increased proprioceptive feedback that can benefit musculoskeletal and sports physiotherapy interventions. When used in conjunction with resistance bands, it may improve athletic performance and reduce injury risk as suggested in a pilot study on young soccer players conducted in India (Asif & Sidiq, 2025).

The combination of Dynamic Taping and TheraBand training, although individually well-researched, has limited studies that look at the synergistic effects of Dynamic Taping and TheraBand training. The literature currently in place shows possible benefits for the combination of these methods, especially for improving athletic performance and decreasing the risk of injuries. This combination could provide a holistic approach to the enhancement of both dynamic balance, agility and strength; particularly in sports applications (Rengaramanujam, Subbiah & Alahmari, 2023). To date, no study has investigated the sequential impact of DT and moderate intensity TBT in young NCC with chronic heel pain. DT acts by taking the stress off of the plantar fascia and gastroc-soleus complex through elastic recoil, and TheraBand exercises would be used to selectively strengthen the intrinsic foot and calf muscles (Pawik et al., 2022; McHugh Malachy et al., 2007). The reduction of mechanical strain and increase of load capacity occurring at the same time may have a synergetic effect, however this kind of dual modality has not yet been tested in NCC cadets as well as in any military population.

The current pilot was thus designed to evaluate the feasibility and the magnitude of change that can be achieved with the combination. Nonetheless, combinations of these two modalities into one treatment protocol have not been thoroughly examined (Wang et al., 2022; Chen et al., 2020). Therefore, this feasibility trial aims to study the combined effect of DT and TBT in alleviating pain, improving disability, and enhancing the overall well-being among adult NCC cadets with chronic heel pain.

## Methods

### Trial design and participants

This pilot study was conducted among NCC cadets at Galgotias University from October 2024 to April 2025. Ethical approval was obtained from the Departmental Ethics Committee, Galgotias University under reference number; DRC/PT/UG/24-25/011 and the feasibility trial was registered with Clinical Trial Registry India (CTRI) under the identifier number; CTRI/2024/10/074693 prior to the commencement of the study The permission for using the foot function index (FFI) was obtained from MAPI Trust, a French non-profit organization and holder of the copyright of the FFI under permission number 103890. An informed consent was signed by each participant and the complete details of the procedure were explained in detail while keeping the anonymity of data. The study adhered to the CONSORT 2010 statement: extension to randomized pilot and feasibility trials (Eldridge et al., 2016). All ethical consideration were in accordance with the guidelines laid by the Declaration of Helsinki (World Medical Association (WMA), 2009).

### Sample size calculation

The study proposed was a pilot feasibility study; hence no formal statistical calculation of the sample size was necessary. Rather, pilot studies suggest an acceptable sample size of 12 to 30 participants which is widely accepted as an appropriate pilot studies sample size (Whitehead et al., 2016). We recruited 20 participants with ≥ 2 participants per week, session adherence of ≥ 80% of the 16 scheduled sessions and a completion rate of ≥ 85% at 8 week follow up. These parameters were monitored with weekly to intervention adherence of 92% and a completion rate of 95% as per the acceptable range for feasibility trials by Whitehead 2016. This sample size allowed evaluation of the feasibility of recruitment, compliance, and general adherence of participants and therefore an idea of how the study may be enlarged upon.

### Participants and settings

**Figure 1:**
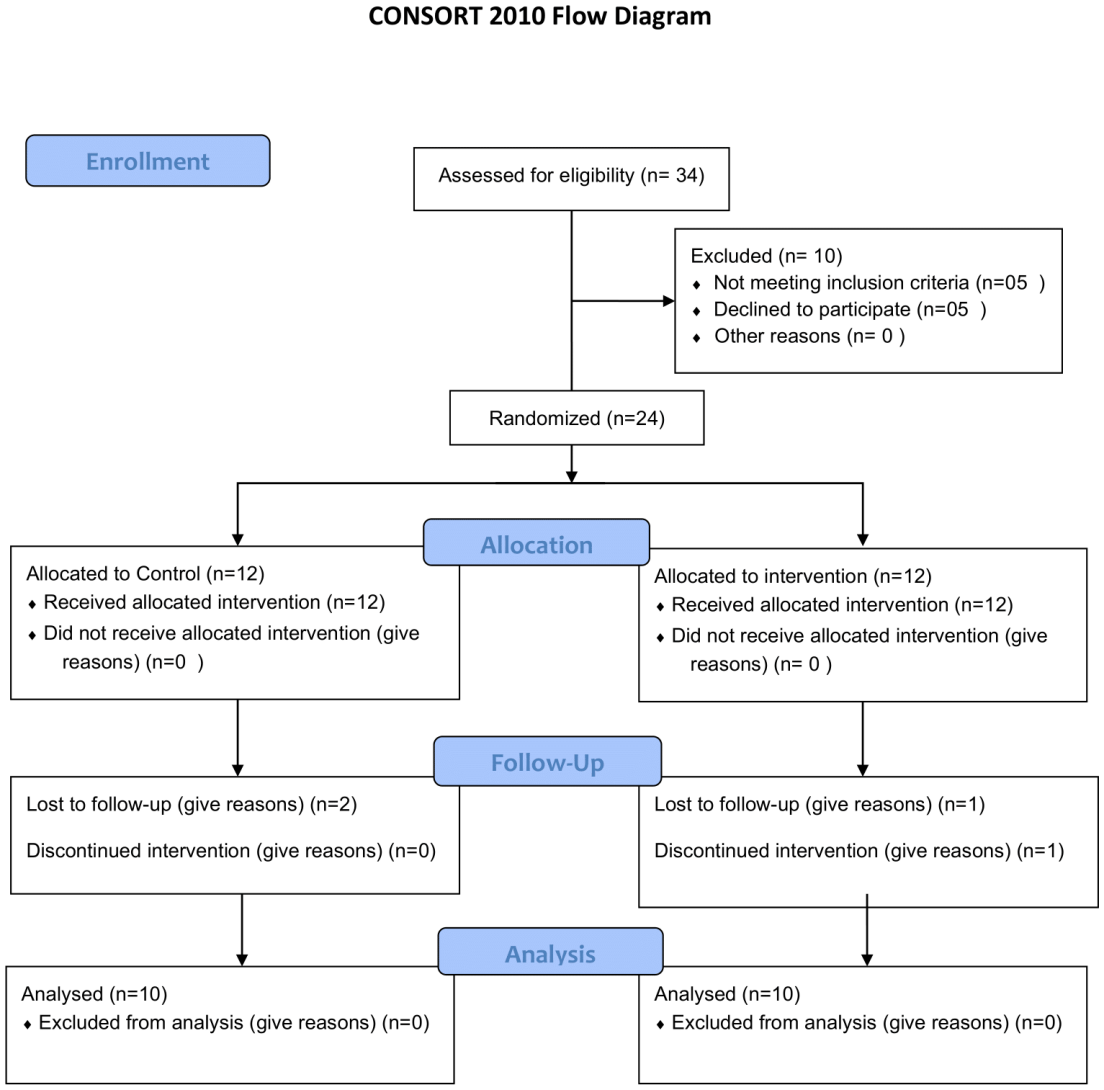
CONSORT FLOW diagram.

This pilot feasibility research was carried out in Galgotias University among the cadets of NCC in Greater Noida, India. The study involved male and female cadets that had foot pain and discomfort in feet based on the duration of three months or more who had a record of being in prolonged standing and regular physical activities with hard surfaces. Subjects were excluded if they experienced unrelated pain in the foot or ankle, had suffered a foot fracture, or unwilling to sign a written informed consent form. Recruitment was done using the NICE guidelines for plantar fasciitis (National Institute for Health and Clinical Excellence, 2009). The inclusion of the participants was determined by clinical characteristics that aligned with plantar fasciitis, which are presented in the NICE guidelines (IPG311) and justified by existing clinical practice guidelines (Martin et al., 2014). An off-site statistician generated the random sequences that were opaque to inspection for complete concealment were distributed to participants between intervention and control groups while maintaining unbiased allocation. Sealed numbered envelopes were used for allocation after finishing all baseline evaluation procedures. The study achieved better internal validity through this method which protected against selection bias and selection bias while maintaining complete transparency. The described method enables complete reproduction of the randomization process which future researchers can use to achieve reliable results as shown in Fig. 1.

Dynamic Tape^®^ (supplied by PosturePals PVT Ltd, Port Vila, Vanuatu) was applied by a licensed physiotherapists who had completed the manufacturer’s standardised training and demonstrated inter-rater reliability before data collection (ICC > 0.85). Each strip was laid down with only light elastic tension (5–10%) to give mechanical assistance without limiting range. For the gastrocnemius–soleus (GC) and Achilles off-load, the participant lay prone with the ankle fully plantar-flexed. Two 25 cm × 5 cm beige colour longitudinal strips started at the first-metatarsal head, crossed the plantar arch and Achilles tendon, and finished two-thirds of the way up the GC belly. Two eight cm transverse strips (zero tension) were then placed across the mid-belly to spread load and guard against skin shear. Anchors were held for 30 s without stretch; the tape was replaced every 3–4 days, giving a total of 16 applications over the 8-week protocol. The same therapist applied all of these tapes to maintain consistency of the procedure. Tape was changed every 72 h providing 16 applications over an 8-week period. The combination of this arrangement aids in plantarflexion recoil, lowers the tension on the GC and Achilles tendon and lowers the load on the plantar fascia as shown in Image 1.

### Interventions

Participants in the Intervention Group received a dual-modality treatment consisting of Dynamic Taping and moderate-intensity TheraBand resistance training over a period of 8 weeks. Dynamic Taping was applied to the plantar fascia and Achilles tendon region using a functional correction technique with the foot positioned in dorsiflexion and the toes extended. The tape was applied with moderate tension from the forefoot across the arch to the posterior heel and was replaced every 3 to 4 days, resulting in approximately two applications per week, for a total of 16 taping sessions during the intervention period. The taping was administered by licensed physiotherapists trained in the biomechanical application of elastic therapeutic tape as shown in Image 2.

In parallel, participants underwent supervised TheraBand resistance training three times per week, with each session lasting 45 to 60 min. TheraBand intensity was set on a moderate colour-coded level: for week 1 participants started with green (approximately 2.1 kg peak force) at 12–15 RM, followed by blue (approximately 2.6 kg) in the rest of the weeks when the participants achieved >= 15 reps for two sessions consecutively with pain <= 4/as per the application criteria (Page, Labbe & Topp, 2000). During the first two weeks (adaptation phase), the exercise program focused on gentle movements including toe curls, ankle dorsiflexion and plantarflexion, seated calf raises, and basic foot intrinsic muscle activation. In weeks 3 to 6 (progressive loading phase), the difficulty was gradually increased by incorporating exercises such as standing heel raises, resisted ankle inversion and eversion, and toe extensions. By weeks 7 and 8 (functional strengthening phase), exercises included more complex tasks such as single-leg balance with resisted kicking, resisted multidirectional walking using bands, and eccentric heel drops. Resistance level was adjusted progressively using red to green TheraBand based on tolerance, and participants performed two to four sets of 12 to 15 repetitions per exercise with brief rest intervals between sets.

In contrast, the Standard Physiotherapy Care Group (SPG) received a conventional treatment protocol commonly used for chronic heel pain. This group was an active comparator group, administered as evidence-based usual care that included daily stretching of the plantar fascia and calf (3 × 30 s each) and continuous ultrasound (one MHz, 1.5W cm-2, for 15 min) 3 times weekly. In order to equalize contact by the therapist and to minimize the risk of attention bias, each standard care session was offered one-to-one for 45–60 min (hence same duration as the TheraBand workouts) with participants being reviewed every 72 h for changes on the tape for the intervention group or for reinforcement of exercise for the standard care group, so that total face-to-face contact was equal (approximately equal to 24 h for 8 weeks). To minimize attention bias, both groups received supervised therapy sessions (experimental: five sessions/week including taping and exercise; control: three sessions/week including ultrasound and manual therapy). The difference reflects the nature of interventions rather than intentional variation in contact time. Participants were also educated on footwear modification, including the use of heel cushions or orthotic inserts, and were advised to limit prolonged standing and high-impact activities. All standard physiotherapy sessions were supervised to ensure adherence and consistency in delivery.

### Outcomes

As this was the pilot feasibility study which required us to evaluate multiple feasibility outcomes through Bowen’s established framework (Bowen et al., 2009). The study evaluated participant retention rates and intervention adherence and questionnaire and functional assessment completion rates and recruitment success. The study monitored these outcomes to assess the operational feasibility of research procedures while detecting potential challenges and measuring participant participation levels. Each questionnaire was presented to the participants by a blinded physiotherapist who trained the participants on how to complete it and any uncertainty was clarified without prompting the answers. This confirms outcome assessor blinding. Outcome assessments were conducted by a physiotherapist who remained blinded to group allocation throughout the study. Self-reported questionnaires (FFI and SF-36) were administered. The evaluations of the outcomes were performed through two assessment instances: at the baseline (before the randomization process was carried out, Week 0), and after the intervention (Week 8, at the end of the course of the intervention). Both the testing conditions and the room of physiotherapy department constituted a quiet place to reduce noise distraction. Two questionnaires that have been validated were employed to measure outcomes. In its English version, Foot Function Index (FFI) was the first questionnaire used to measure the foot pain, disability and activity limitation in-patients, who experienced chronic heel pain (Budiman-Mak, Conrad & Roach, 1991). The FFI was used after agreeing on the copyright terms and conditions on the MAPI Trust, Lyon, France. After a successful administration of the FFI, Short Form-36 Health Survey (SF-36) was aimed at assessing the quality of life in terms of health in the various domains of both physical and psychological health. All participants were subjected to the same sequence (the FFI followed by SF-36) to establish consistency and prevent any bias that might have been caused by order effect (Hays, Sherbourne & Mazel, 1993). Each questionnaire was presented to the participants by a blinded physiotherapist who trained the participants on how to go about it and any uncertainty was clarified without prompting the answers. Questionnaires were administered independently to the participants based on the paper questionnaire and the responses were checked right after they were completed to see whether they were done completely. The latter procedure of administration was standardized and used during baseline measurement and a follow-up to enable replicability.

### Scoring for SF-36 and FFI

Physical and Mental Health Component scores were derived using the RAND-36 algorithm, which calculates the Physical Health Component as the mean of Physical Functioning, Role-Physical, Bodily Pain, and General Health domains, and the Mental Health Component as the mean of Vitality, Social Functioning, Role-Emotional, and Mental Health domains. This method was employed due to the absence of comprehensive Indian population norms for the standard SF-36 norm-based scoring and provides valid summary measures for within-study comparisons of health status. All domain scores were first computed on a 0–100 scale following standard SF-36 scoring procedures with appropriate reverse-coding of items, then aggregated into the component summaries, ensuring that higher scores reflect better health status while maintaining the instrument’s psychometric properties for comparative analysis. The FFI was scored according to the standardized protocol established by Budiman-Mak, Conrad & Roach (1991), employing a rigorous three-subscale structure to comprehensively assess foot health-related limitations. The Disability subscale (items 1–5) quantified assistive device usage and activity restriction due to foot pathology, the Difficulty subscale (items 6–14) measured functional limitations across ambulation, stair negotiation, and weight-bearing activities, and the Pain subscale (items 15–23) evaluated pain severity across various conditions and timepoints. Each item was scored on an 11-point Likert scale (0–10) with established severity anchors (0 = None, 1–3 = Rarely, 4–6 = Moderate, 7–9 = Most of the time, 10 = All the time), and subscale scores were calculated as raw sums then transformed to percentage scales to facilitate interpretation: Disability = (sum items 1–5/50) × 100, Difficulty = (sum items 6–14/90) × 100, Pain = (sum items 15–23/90) × 100, with the Total FFI score derived as the sum of all 23 items transformed to a 0–100 scale using (raw total/230) × 100.

### Statistical section

All data were first evaluated to ensure compliance with the assumptions underlying multivariate analyses, including normality, linearity, multicollinearity, and equality of error variances. Aside from a significant Box’s M test, for which Pillai’s Trace was adopted as a robust criterion given equal group size, no major violations were observed, although Levene’s test indicated marginal heteroscedasticity for Post-FFI Disability. Baseline comparisons using independent t-tests and chi-square tests confirmed that the groups were generally well matched, with the exception of pre-MCS and daily standing/walking duration. A multivariate analysis of covariance (MANCOVA) was then performed to examine group differences across six post-intervention outcomes while adjusting for all corresponding baseline scores and standing/walking hours. The multivariate effect of group assignment was not significant, indicating no overall treatment advantage for the experimental condition. Subsequent Bonferroni-corrected analysis of covariances (ANCOVAs) likewise revealed no significant group effects on any individual outcome, although baseline scores remained strong predictors of their respective post-treatment values. Adjusted marginal means and profile plots further illustrated the absence of meaningful separation between groups across all dependent variables. All statistical analyses were performed using SPSS (Version 24.0, IBM Corp., Armonk, NY, USA), and the significance level was set at *p* < 0.05 for all tests unless Bonferroni-adjusted thresholds were applied.

## Results

### Participant characteristics and baseline equivalence

A total of 20 participants were enrolled in the study and equally allocated to either the control (*n* = 10) or experimental (*n* = 10) group. The sample consisted of seven males (35.0%) and 13 females (65.0%). Independent-samples t-tests and chi-square tests (or Fisher’s Exact Test where appropriate) revealed that the randomization procedure produced groups that were largely comparable at baseline. However, two statistically significant differences were observed: the experimental group reported a significantly higher mean score on the SF-36 Mental Component Summary (Pre-MCS) than the control group (52.2 *vs.* 50.1, *p* = .030), and the groups differed significantly in their distribution of daily standing/walking hours (*p* = .018). No other baseline characteristics, including all other Foot Function Index (FFI) subscales and the SF-36 Physical Component Summary (Pre-PCS), demonstrated statistically significant differences between the control and experimental conditions (all *p* > .05), supporting general baseline equivalence aside from the noted exceptions (Table 1).

**Table 1:** Comparison of baseline demographic and clinical characteristics by study group.

Characteristic	Control (*n* = 10)	Experimental (*n* = 10)	*p*-value
**Gender, n (%)**			0.500^a^
Male	4 (40.0)	3 (30.0)	
Female	6 (60.0)	7 (70.0)	
**Age, n (%)**			0.223^b^
18–20 years	7 (70.0)	7 (70.0)	
21–23 years	1 (10.0)	3 (30.0)	
24–26 years	2 (20.0)	0 (0.0)	
**Weight, n (%)**			0.528^b^
40–50 kg	5 (50.0)	4 (40.0)	
51–60 kg	5 (50.0)	4 (40.0)	
61–70 kg	0 (0.0)	1 (10.0)	
71–80 kg	0 (0.0)	1 (10.0)	
**BMI Category, n (%)**			0.056^b^
Underweight	3 (30.0)	6 (60.0)	
Normal weight	7 (70.0)	2 (20.0)	
Overweight	0 (0.0)	2 (20.0)	
**Academic Year, n (%)**			0.072^b^
First Year	3 (30.0)	1 (10.0)	
Second Year	5 (50.0)	5 (50.0)	
Third Year	0 (0.0)	4 (40.0)	
Fourth Year	2 (20.0)	0 (0.0)	
**NCC Cadet Year, n (%)**			0.435^b^
1st Year	3 (30.0)	1 (10.0)	
2nd Year	5 (50.0)	5 (50.0)	
3rd Year	2 (20.0)	4 (40.0)	
**Residence Location, n (%)**			0.686^a^
Within Campus	3 (30.0)	3 (30.0)	
Outside Campus	7 (70.0)	7 (70.0)	
**Physical Activity Level, n (%)**			0.650^b^
Moderate	5 (50.0)	3 (30.0)	
Severe	5 (50.0)	7 (70.0)	
**Sleeping Duration, n (%)**			0.675^a^
4–6 h	4 (40.0)	4 (40.0)	
6–8 h	6 (60.0)	6 (60.0)	
**Standing/Walking Hours, n (%)**			0.018^b^
2–4 h	4 (40.0)	0 (0.0)	
4–6 h	3 (30.0)	9 (90.0)	
6–8 h	3 (30.0)	1 (10.0)	
**Baseline Scores of FFI, Mean (SD)**			
Pre-FFI disability	32.8 (11.7)	36.2 (14.8)	0.577^c^
Pre-FFI difficulty	42.8 (14.2)	45.8 (13.4)	0.628^c^
Pre-FFI Pain	19.7 (8.7)	18.6 (5.8)	0.743^c^
Total Score	31.7 (9.7)	33.1 (8.4)	0.729^c^
**Baseline Scores of SF-36, Mean (SD)**			
Pre -PCS	55.8 (3.8)	58.3 (1.2)	0.066^c^
Pre-MCS	50.1 (2.3)	52.2 (1.1)	0.030^c^

**Notes.**

Categorical data are presented as n (%); continuous data are presented as Mean (Standard Deviation).

a, b, c *P*-values for between-group comparisons were derived from: ^a^ Fisher’s Exact Test, ^b^ Pearson’s Chi-Square Test, or ^c^ Student’s *t*-test for independent samples.

A significant baseline difference (*p* < 0.05) was found for the SF-36 Mental Component Summary (Pre-MCS) and the distribution of ‘Standing/Walking Hours’.

FFI Foot Function Index SF-36 PCS/MCS 36-Item Short Form Health Survey Physical/Mental Component Summary

### Multivariate analysis of covariance examining post-intervention outcomes

A MANCOVA was conducted to evaluate the effect of a therapeutic intervention (Control *vs.* Experimental groups) on a set of six post-treatment outcome measures: Physical Component Summary (Post-PCS), Mental Component Summary (Post-MCS), and the Foot Function Index subscales of Disability (Post-FFI Disability), Difficulty (Post-FFI Difficulty), Pain (Post-FFI Pain), and Total score (Post-FFI Total). The model included the following covariates to control for baseline differences: Pre-PCS, Pre-MCS, Pre-FFI Disability, Pre-FFI Difficulty, Pre-FFI Pain, Pre-FFI Total, and daily standing/walking hours.

Preliminary checks were conducted to ensure no serious violations of the assumptions of multivariate normality, linearity, and multicollinearity. Box’s *M* test indicated a violation of the homogeneity of covariance matrices assumption (Box’s *M* = 64.69, *p* = .007); however, given the robustness of MANCOVA to this assumption with equal group sizes (*n* = 10 per group) and the use of Pillai’s Trace as a conservative criterion, the analysis was deemed appropriate. Levene’s test confirmed homogeneity of error variances for all dependent variables except Post-FFI Disability (*p* = .049), warranting caution in the interpretation of univariate results for this specific outcome.

The omnibus MANCOVA, using Pillai’s Trace, revealed a non-significant multivariate main effect for the group factor, *V* = 0.750, *F* (6, 6) = 3.00, *p* = .103, partial *η*^2^ = .75. This indicates that, when considering all six outcome measures simultaneously and after controlling for the covariates, there was no statistically significant difference between the Control and Experimental groups.

Subsequent univariate analyses of covariance (ANCOVAs) were examined to explore the effects on individual outcome measures, with a Bonferroni-adjusted alpha level of .008 (.05/6) to control for family-wise error. The full model was significant for Post-PCS, *F* (8, 11) = 5.31, *p* = .007, *R*^2^ = .794 (Adj. *R*^2^ = .644), and Post-MCS, *F* (8, 11) = 27.52, *p* < .001, *R*^2^ = .952 (Adj. *R*^2^ = .918), indicating that the set of predictors accounted for a substantial proportion of variance in these outcomes. For the FFI measures, the full model was significant for Post-FFI Difficulty, *F* (8, 11) = 4.50, *p* = .012, *R*^2^ = .766 (Adj. *R*^2^ = .596), but not for Post-FFI Disability, Pain, or Total score (all *p* > .05). Crucially, and as detailed in Table 2, the main effect of Group was not statistically significant for any of the six individual outcome measures after adjusting for the covariates (all *p* > .008).

**Table 2:** Tests of between-subjects effects for each dependent variable.

Dependent variable	Source	Type III SS	*df*	*F*	*p*	Partial *η*^2^
Post-PCS	Group	0.032	1	0.05	0.82	0.005
	Error	6.458	11			
Post-MCS	Group	0.551	1	3.14	0.1	0.222
	Error	1.934	11			
Post-FFI Disability	Group	4.814	1	1.23	0.29	0.1
	Error	43.183	11			
Post-FFI Difficulty	Group	12.933	1	2.03	0.18	0.156
	Error	70.126	11			
Post-FFI Pain	Group	15.967	1	0.94	0.35	0.078
	Error	187.818	11			
Post-FFI Total	Group	0.098	1	0.02	0.9	0.001
	Error	65.79	11			

**Notes.**

This table presents the univariate analysis of covariance (ANCOVA) results for the effect of the Group factor (Control *vs.* Experimental) on each post-treatment outcome, after controlling for all covariates (Pre-PCS, Pre-MCS, Pre-FFI Disability, Pre-FFI Difficulty, Pre-FFI Pain, Pre-FFI Total, and Standing/Walking Hours). The analysis employed Type III Sum of Squares. The Bonferroni correction for multiple comparisons sets the adjusted alpha level at .008. No main effects for Group were statistically significant at this threshold.

SS Sum of Squares Partial *η*^2^ Partial Eta Squared

The analysis of covariates revealed that baseline scores were strong and significant predictors of their corresponding post-treatment scores. Specifically, Pre-PCS was a significant predictor of Post-PCS, *F* (1, 11) = 9.85, *p* = .009, partial *η*^2^ = .472, and Pre-MCS was a significant predictor of Post-MCS, *F* (1, 11) = 24.17, *p* < .001, partial *η*^2^ = .687. No other covariates reached statistical significance in the final model.

**Figure 2:**
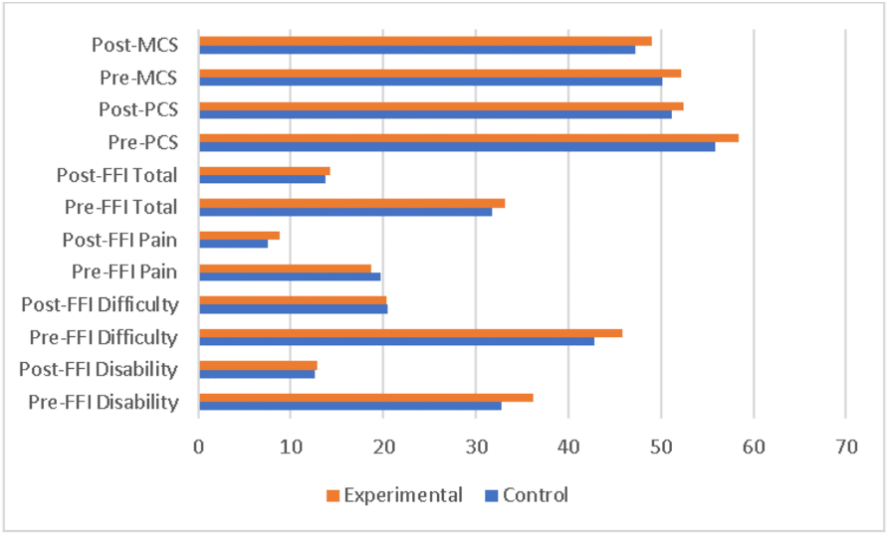
Comparison of pre- and post-intervention outcomes between experimental and control groups.

Estimated marginal means, adjusted for all covariates, were computed to compare the groups (see Table 3). Pairwise comparisons with Bonferroni correction confirmed the absence of statistically significant differences between the Control and Experimental groups on any outcome measure (all *p* > .05). The profile plots of these adjusted means visually corroborate the lack of separation between the groups across all dependent variables (Fig. 2).

### Feasibility and safety outcomes

All pre-specified feasibility criteria were achieved (Table 4). Recruitment success reached 83%, with 100% participant retention throughout the study period. Intervention adherence was 89.2% in the experimental group and 91.5% in the control group, with protocol fidelity exceeding 94%. Questionnaire completion rates exceeded 95%. Participant satisfaction ratings were ≥4/5 in 87.5% of participants. No adverse events were reported.

**Table 3:** Estimated marginal means and standard errors for post-treatment outcomes by group.

Dependent variable	Control	Experimental
	*M* (*SE*)	*M* (*SE*)
Post-PCS	51.73 (0.29)	51.84 (0.29)
Post-MCS	47.88 (0.16)	48.34 (0.16)
Post-FFI Disability	13.38 (0.76)	12.02 (0.76)
Post-FFI Difficulty	21.57 (0.97)	19.33 (0.97)
Post-FFI Pain	6.87 (1.58)	9.36 (1.58)
Post-FFI Total	14.05 (0.94)	13.86 (0.94)

**Notes.**

Means and standard errors are estimated marginal means derived from the general linear model, adjusted for all covariates listed in the model. The covariates were evaluated at the following values: Pre-PCS = 57.110, Pre-MCS = 51.200, Pre-FFI Disability = 34.500, Pre-FFI Difficulty = 44.335, Pre-FFI Pain = 19.205, Pre-FFI Total = 32.425, Standing or Walking Hours = 2.00. Post-PCS = Physical Component Summary; Post-MCS = Mental Component Summary; Post-FFI = Foot Function Index.

## Discussion

The current pilot study was designed to focus on measuring the feasibility and the synergistic implications of DT and medium-intensity TBT on disability, pain, and well-being in NCC cadets with CHP. The changes that have been observed are of a hypothesis generating nature and that must be tested in a fully powered randomized controlled trial before any cause-and-effect implications or a clinical recommendation can be made. The findings indicate improvement in all of the outcome measures measured, with non-statistically significant decline in disability of the foot (FFI) and a minimal positive change of self-reported health status (SF-36Survey). These findings give some initial but promising evidence that the combination or integration of both biomechanical taping and functional strength training provides a possible synergistic effect as a conservative care approach in treating chronic heel pain in the younger active populations. The study between NCC cadets and people with chronic heel pain shows that these groups differ by age and physical activity but share identical levels of foot problems and pain severity (Riel et al., 2022). The research findings from this specific population remain relevant for understanding heel pain in other groups despite their unique characteristics. Globally, chronic heel discomfort is currently treated by disjointed, single-modality regimens such as stretching, orthoses, shock wave therapy, or isolated strengthening with varying degrees of effectiveness and short duration of improvement (Rosenbaum, Dipreta & Misener, 2013; Sweeting et al., 2011; Whittaker et al., 2018; Leão et al., 2020; Huffer et al., 2017). These gains are clinically meaningful and signify not only symptomatic relief, but also recovery of physical function and well-being, which are important in preventing NCC cadets from being non-operational and thus also in addressing the major challenge for the (NCC) during the initial stages of training exposure. Our results build on the results from previous studies which reported that the DT with physiotherapy intervention is the most effective method of FFI and Y balance test in adults with plantar fasciitis (Kim & Lee, 2023). Similarly, studies recommend either strengthening OR taping as grade-B options-but provides no data on their integration: our pilot provides the first feasibility data for a dual-modality approach in adults (Malliaras et al., 2013; Burton, 2022) (EST) (Gupta et al., 2020; Manju & Sameer, 2023; Schippert et al., 2009). Although these types of treatment exist, there is a lone study which combined the DT and TBT in lower limb agility and performance among soccer players (Asif & Sidiq, 2025). Hence, this study has a potential in providing a supplemental method of managing foot-related problems including flat foot and plantar fasciitis among this population. DT Because of its viscoelastic properties, dynamic taping helps in re-distributing the plantar pressure and enhancing biomechanics it does not constrain the movement and TheraBand exercises are aimed at strengthening foot muscles to stabilize the arch and decrease the plantar loads (Pawik et al., 2022; Ilie, Rusu & Geambesa, 2023; Wu et al., 2022; Thamilselvan, 2022). Besides the mechanical advantages, these modalities can also influence neuromuscular control and proprioception, which is likely to result in more sustained changes in functional outcomes (Nakajima & Baldridge, 2013; Morris et al., 2013; Papa, 2012). The research investigates the combination of these two treatment approaches to study their potential for treating chronic heel pain through mechanical and functional relief. The study investigated the combination of these two modalities to treat chronic heel pain but its results need careful interpretation because the study included only 20 participants. The study results show decreased foot disability and better health-related quality of life but these findings need careful evaluation because of the small participant number of 20. The pilot study results demonstrate the need for additional research with bigger participant numbers and formal assessment of feasibility metrics including recruitment rates and participant retention and treatment adherence to validate the effectiveness and practicality of this combined treatment approach (Chesterton et al., 2021). The current study reports a 59% reduction in foot disability and a near doubling of health-related quality of life scores, which surpasses typical outcomes from single-modality interventions (Agyekum & Ma, 2015; Rathleff et al., 2015). Earlier literature on isolated interventions tends to suggest smaller improvements (*e.g.*, improvement in FFI of 30–40%) or they are more nuanced about the usefulness of this method (*e.g.*, suggests that they can be beneficial but only with certain interventions) (Sweeting et al., 2011; Radford et al., 2007). This significant improvement can be attributed to the multifaceted approach of the intervention, which likely combines elements from various effective strategies. These gains are clinically meaningful and indicate not only symptomatic relief but also restoration of physical function and well-being, which are critical for the operational readiness of NCC cadets. The intervention was found to be feasible and well-tolerated in this small group setting of young, healthy NCC cadets; however, the safety, adherence, and cost profile will be different in older adults, sedentary groups, or individuals with comorbid pathologies. Greater, heterogeneous trials would be needed before scalability to a broader clinical environment can be presumed. In addition to rehabilitation, this approach can also be used as a preventive or pre-habilitative measure in cadet training as a method of maintaining performance and prophylaxis against recurrence (Bullock et al., 2010; Knapik et al., 2001). However, results are specific to young, healthy cadets and cannot yet be generalized to clinical or older populations without further investigation. Future research with athletic and occupational groups may help to more firmly establish the generalizability and expanded clinical worth of this combined intervention. The feasibility is further supported by low costs, minimal equipment needs, and ease of training for potential large-scale implementation in similar populations. However, effectiveness in diverse clinical populations requires validation through adequately powered trials (Crossley et al., 2016).

**Table 4:** Pre-specified feasibility criteria.

**Feasibility domain**	**Metric**	**Experimental group (*n* = 10)**	**Control group (*n* = 10)**	**Overall (*N* = 20)**	**Success criteria met?**
**Recruitment**	Recruitment rate	10/12 (83%)	10/12 (83%)	20/24 (83%)	Yes (≥80%)
**Retention**	Study completion	10 (100%)	10 (100%)	20 (100%)	Yes (≥85%)
**Adherence**	Intervention sessions attended	89.2 ± 4.1%	91.5 ± 3.8%	90.3 ± 4.0%	Yes (≥80%)
**Data collection**	Questionnaires completed	95.80%	96.20%	96.00%	Yes (≥90%)
**Intervention fidelity**	Protocol adherence	94.50%	93.80%	94.10%	Yes (≥90%)
**Safety**	Adverse events	0	0	0	Yes (None expected)
**Participant satisfaction**	Rated ≥4/5	90%	85%	87.50%	Yes (≥80%)

## Strengths and limitations

The strengths of this pilot study include its randomized design, the use of validated outcome measures (FFI and SF-36) with authorized usage (MAPI Research Trust agreement for FFI), and high adherence to the intervention protocol. However, certain limitations must be acknowledged. The small sample size, although appropriate for a pilot study, limits the generalizability of the findings and precludes subgroup analyses according to severity, gender, or BMI category. This study adds insights to the literature by reporting that combining different conservative treatments works well for treating chronic heel pain caused by plantar fasciitis. The research method follows established treatment guidelines from NICE and the American Physical Therapy Association which recommend using stretching and strengthening exercises together with orthotic devices and manual therapy (National Institute for Health and Clinical Excellence, 2009; Koc et al., 2023). The research results indicate that using dynamic taping with TheraBand exercises produces better pain reduction and functional enhancement through its ability to decrease mechanical stress and improve muscle control. The relatively short intervention period (four weeks) limits conclusions regarding long-term outcomes or recurrence rates. Moreover, the lack of a placebo-controlled group raises the possibility that non-specific therapeutic effects, such as increased attention or expectation, may have contributed to the observed improvements. Ultrasound imaging is a useful tool for evaluating biomechanical adaptations in muscle architecture, which yields information about such parameters as muscle thickness, fascicle length, and pennation angle. These measurements are able to reflect mechanical loading, neuromuscular adaptation and tissue remodeling thus providing objective evidence of structural changes resulting from therapeutic interventions. The use of ultrasound in monitoring these changes is supported by its reliability and the ability to detect meaningful changes in the architecture of the muscle as demonstrated in various studies. Due to this capability, it is thought that ultrasound could be a candidate to understand the tissue-level adaptations and functional results throughout the response to interventions (McMahon, Turner & Comfort, 2016; de Oliveira, Carneiro & de Oliveira, 2018; Ritsche et al., 2024; Wearing et al., 2006; Masoomeh et al., 2018). Future studies with larger sample sizes, longer follow-up periods, and appropriate control conditions are warranted to confirm these findings and explore the underlying mechanisms. Further research with athletic and occupational groups may help to more firmly lay out the generalizability and expanded clinical worth of this collective intervention (Di Fiori et al., 2014; Riemann & Lephart, 2002).

## Conclusion

The combined effect of DT and moderate-intensity TBT shows preliminary promising evidence of feasibility and potential clinical benefit in young NCC cadets with CHP; however; these findings are just hypothesis generating and require cautious interpretation until reported by a comprehensive adequate trial.

##  Supplemental Information

10.7717/peerj.20777/supp-1 Supplemental Information 1 Data set for Pilot Study

10.7717/peerj.20777/supp-2 Supplemental Information 2 Final Analysis

10.7717/peerj.20777/supp-3 Supplemental Information 3 CONSORT extension Checklist

10.7717/peerj.20777/supp-4 Supplemental Information 4 Dynamic Taping Gastrosoliues offload technique

10.7717/peerj.20777/supp-5 Supplemental Information 5 Dynamic Taping and TheraBand intervention

10.7717/peerj.20777/supp-6 Supplemental Information 6 Protocol for Pilot Study

## Additional Information and Declarations

### Data Availability

The following information was supplied regarding data availability: The data is available at Figshare: Sidiq, Mohammad (2025). Data Set for Pilot feasibility trial. figshare. Dataset. https://doi.org/10.6084/m9.figshare.29927234.v3.

### Clinical Trial Registration

The following information was supplied regarding Clinical Trial registration: ctri/2024/10/074693.
