# Supplementary figures and images for: Effects of dynamic taping and moderate-intensity TheraBand training versus usual care on pain, disability, and well-being in adult National Cadet Corps with chronic heel pain: a pilot and feasibility trial
Source: PeerJ. 2026 Mar 6;14:e20777. doi: 10.7717/peerj.20777 (PMC12970306; doi:10.7717/peerj.20777)

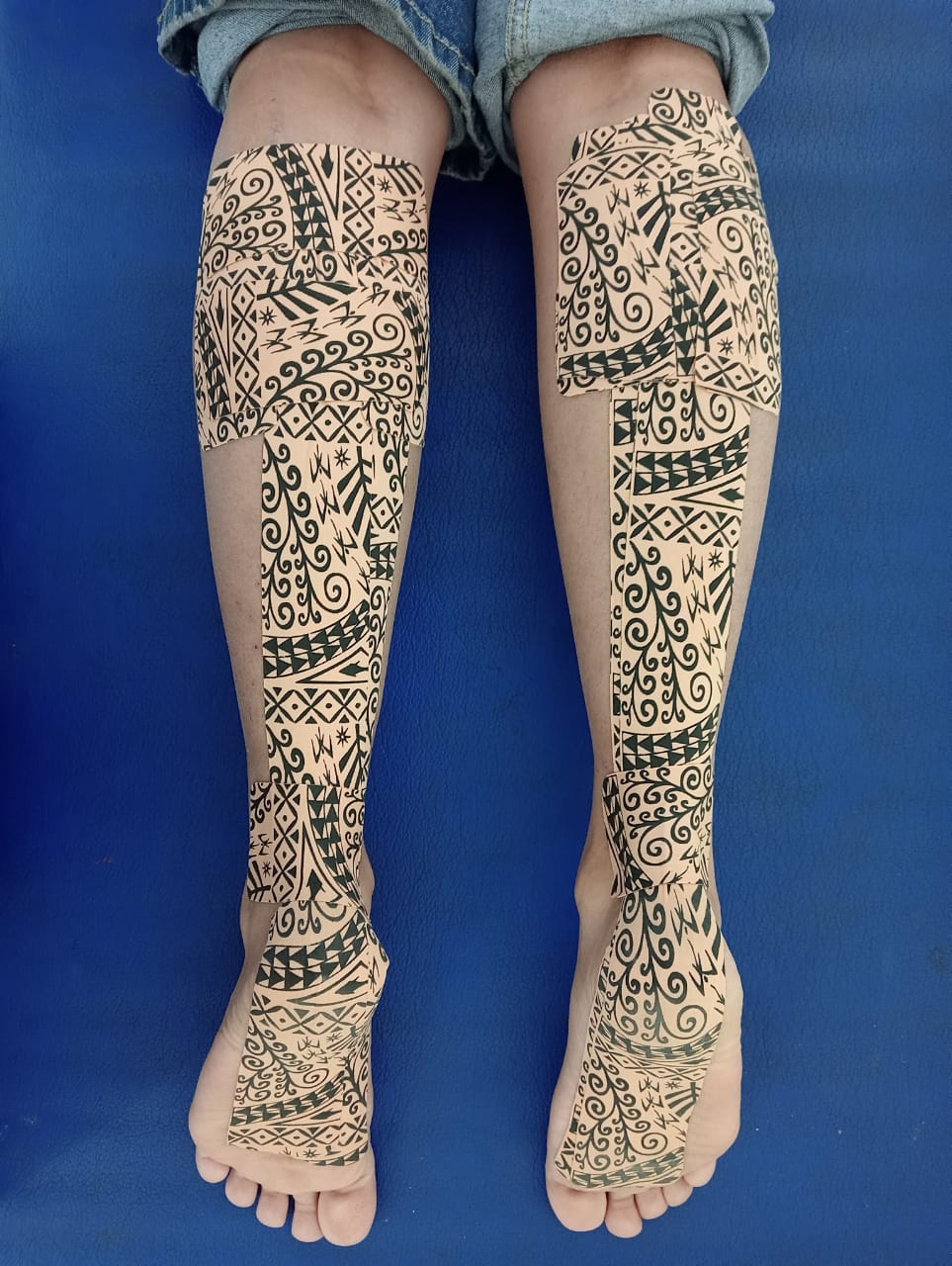

Supplement: Supplemental Information 4 [file peerj-14-20777-s004.tiff]

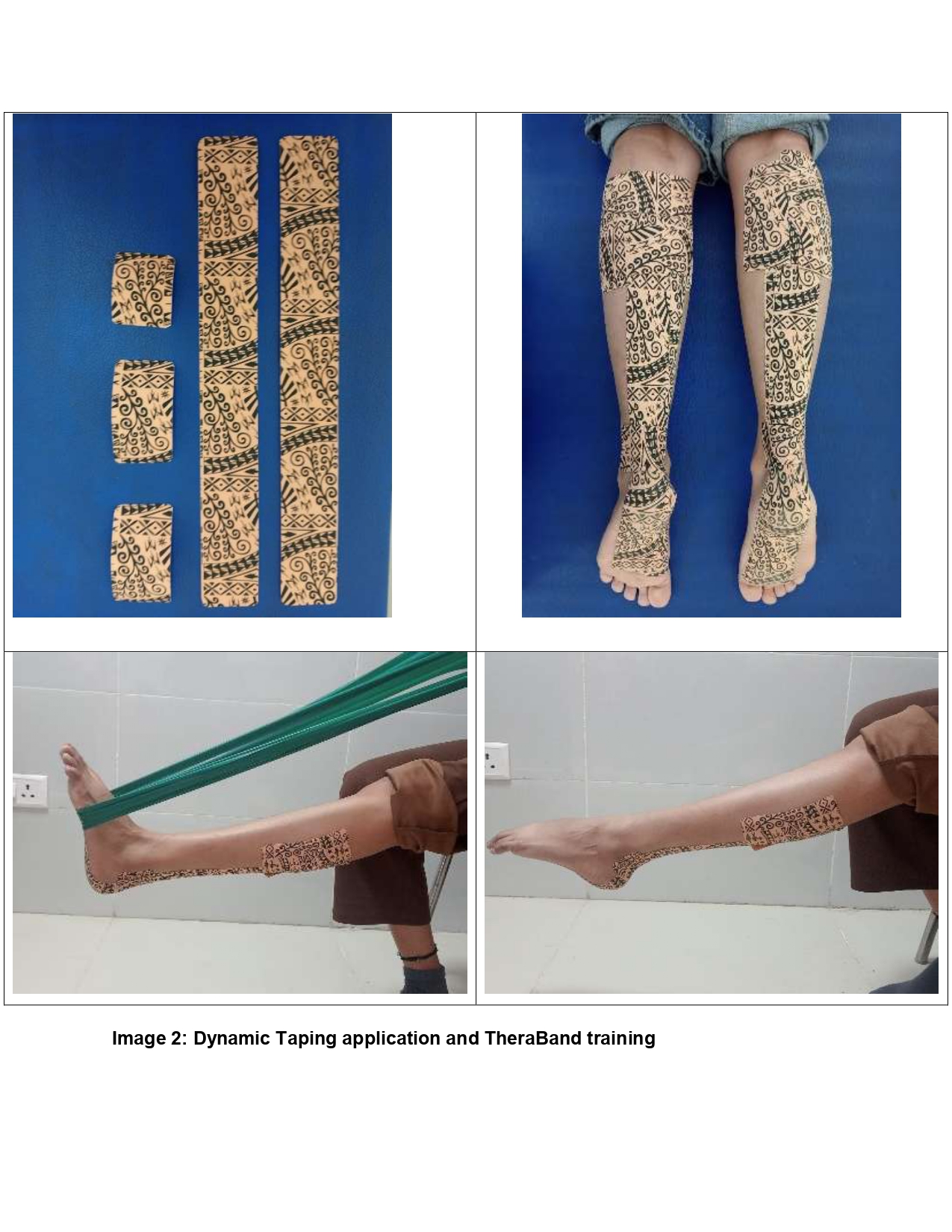

Supplement: Supplemental Information 5 [file peerj-14-20777-s005.jpg]
