# Supplementary material for: Effects of dynamic taping and moderate-intensity TheraBand training versus usual care on pain, disability, and well-being in adult National Cadet Corps with chronic heel pain: a pilot and feasibility trial
Source: PeerJ. 2026 Mar 6;14:e20777. doi: 10.7717/peerj.20777 (PMC12970306; doi:10.7717/peerj.20777)
Supplement: Supplemental Information 6 [file peerj-14-20777-s006.docx]

**TITLE:** Effects of Dynamic Taping and Moderate-Intensity TheraBand Training versus Standard Physiotherapy Care on Pain, Disability, and Well-Being in Adult National Cadet Corps with Chronic Heel Pain: a pilot and feasibility trial

**INTRODUCTION**

Chronic heel pain (CHP) is a significant cause of discomfort and impairment that affects the quality of life of numerous active adult individuals.(1). Heel pain is a common foot condition known by various names, including plantar fasciitis, jogger's heel, tennis heel, and policeman's heel. These terms generally refer to inflammation or irritation of the heel's soft tissues. Chronic plantar fasciitis, known as major cause of the chronic heel pain, is recognized as the most common foot condition, affecting both young adults and older individuals.(2,3) frequently associated with persistent symptoms and functional limitations affecting both the athletic and nonathletic populations.(4) Plantar fasciitis is a chronic degenerative condition that results in medial plantar heel pain and accounts for approximately 1 million physician visits annually. Patients typically experience the most intense pain with their initial steps in the morning or after extended periods of standing.(5–7). As we know that the plantar fasciopathy is a common cause of heel pain, but chronic heel pain (CHP) can also result from Achilles tendinitis, obesity, heel fat pad syndrome (HFPS), and lumbar radiculopathy. Each condition involves different underlying issues, such as inflammation, mechanical stress, or nerve irritation, which need targeted treatments(8,9).Overweight and obesity increases musculoskeletal stress , affecting gait, balance, and joint loads with severity worsening as BMI rises. For instance, BMI over 30 exacerbate the biomechanical challenges , heightening the risk of conditions like heel pain, plantar fasciitis, OA.(10,11). The 1990 US National Health Interview Survey, which included 119,631 people over the age of 18, found that 24% of them reported foot problems.(12). The Global Burden of Disease (GBD) study reported that in 2020, approximately 494 million people were affected by musculoskeletal disorders. Projections suggest a 123% increase in the global burden of these conditions by 2050, with a significant impact anticipated in low and middle-income countries.(13). Research indicates that heel pain is prevalent in middle-aged and older adults, with estimated rates ranging from 3.6% to 7.3% in adults aged 18 and older, and 9.6% to 11.1% in middle-aged and older populations.(14,15) Occupations involving prolonged standing and walking, such as those of Policeman, NCC Cadets, nurses, security guards, and postal workers, can increase the risk of developing heel pain due to the continuous strain and pressure on the feet.(16). Biomechanical dynamic tape (DT) and kinesiology tape (KT), is used in sports medicine to support and stabilize muscles, joints, and ligaments, particularly for ankle and foot injuries. It helps improve movement and reduce pain during recovery.(17). Dynamic Tape uses its elastic properties to absorb and release energy (bungee effect) when applied to muscles, providing immediate support and relief. This can aid in managing symptoms while waiting for more targeted treatment.(18). TheraBand exercise intervention for individuals with Moderate Intellectual Disability (MID) has significant enhancements in muscle strength and motor development.(19) Thera-Band muscle strengthening exercises effectively improve musculoskeletal conditions , lower limb function in hemiplegic stroke patients aiding in their rehabilitation and mobility.(20)

**Rationale:**

Extensive research on foot pain and foot health indicate that individuals aged 18 years and above who stand for more than 6 hours daily and who wear inappropriate or low-quality footwear including hard leather shoes have an increased risk of developing musculoskeletal disorders such as chronic heel pain, plantar fasciitis, and ankle pain.(1,5,7,8,14).Heel pain is a prevalent condition that significantly impacts quality of life and functional performance, affecting both athletic and non-athletic populations. For National Cadet Cops (NCC) who often engage in rigorous training involving prolonged standing, marching, and physical activities understanding the prevalence and management of heel pain is crucial.(4)NCC cadets are regularly subjected to physical demands that can exacerbate or trigger heel pain, including plantar fasciitis and other related conditions. Given the physical intensity and duration of their activities these individuals may be at heightened risk for chronic heel pain which could impair their performance, well-being, and overall training efficacy.(12,13).Research into heel pain among NCC cadets is vital for improving their health outcomes, optimizing training effectiveness, and ensuring that the cadets can fulfill their duties effectively without undue physical limitations, therefore the study aims to gain insights on the possible effects of dynamic taping and moderate intensity TheraBand training on CHP among NCC Cadets.

**Objectives**

**Primary -** To evaluate the effects of dynamic taping and TheraBand exercises on pain, disability in CHP among NCC Cadets.

**Secondary** - To examine the possible effects of dt on overall well-being among NCC Cadets

**REVIEW OF LITERATURE**

**Search strategy**

**Condition/population** – Chronic plantar heel pain among young NCC Cadets

**Type of studies included** - A Pilot Study, Clinical trials, Randomized Controlled Trial, Quasi experimental studies, Reviews, Case study.

**Keywords**- Young Adults, Dynamic Taping, Chronic Heel pain, TheraBand training, NCC Cadets, Prolonged standing.

**Databases Searched**- PubMed, Science Direct, Research Gate, Google-Schloar, PEDro database

**Your Searched** – 2014-2024

**Language**- English

**Methodology**

**Design**- A Pilot feasibility trial.

**Setting-** Data will we collected from NCC Cadets of Galgotias University.

**Ethical Approval & Informed Consent**- Ethical approval will be obtained by the Department Ethics Committee, Galgotias University. The protocol of the study will be prospectively registered with Clinical Trial Registry (CTRI), India. Prior permission to use the validated Hindi version of Foot Function Index was obtained by signing a special COA agreement with the MAPI TRUST – a non-profitable research organization ,27 rue de la Villette, 69003 Lyon, France. All participants will be informed that participation is entirely voluntary and they are free to withdraw at any time without any reason.

**Inclusion Criteria**

1. NCC Cadets with CHP more than 3 months duration
2. Cadets with standing time more than 6 Hours more
3. Both males and females will be included in the study.
4. Cadets exercising on hard surfaces or concrete

**Exclusion Criteria**

1. Cadets suffering from any ankle or foot pain
2. Any previous history of foot fracture or deformity.
3. Participants unwilling to sign informed consent.

**Sample Size Calculation**

A total number of 20 NCC Cadets will be recruited for this study as per the rule of thumb for pilot study.

**Procedure**

1. Ethical Approval and Informed Consent Obtain ethical approval from the Departmental Ethics Committee at Galgotias University. Ensure all participants sign an informed consent form, detailing the study’s purpose, procedures, and potential risks. All participants will be informed that participation is entirely voluntary and they are free to withdraw at any time without any reason.

2. Participant Recruitment and Screening

- After screening for the eligibility criteria, potential 20 NCC Cadets with chronic heel pain (CHP) of more than 3 months duration.
- Screen for inclusion criteria: standing time exceeding 6 hours daily, both genders, and exercising on hard surfaces or concrete.
- Exclude individuals with ankle or foot pain unrelated to CHP, history of foot fractures or deformities, and those unwilling to consent.

3. Baseline Data Collection

- Collect demographic data from participants, including age, gender, and duration of CHP.
- Assess baseline foot function and overall well-being using the Foot Function Index (FFI) and SF36 questionnaires.

4. Intervention

Dynamic Taping (DT) Group: Apply dynamic tape to the affected heel to provide support and alleviate pain. Ensure proper taping technique and adjust as necessary based on individual feedback. TheraBand Training Group: Implement a moderate-intensity TheraBand training regimen designed to strengthen the foot and ankle muscles. Ensure exercises are performed correctly and adjust intensity as needed based on participant feedback.

5. Intervention Duration and Monitoring

Conduct interventions for a predefined period (e.g., 6 weeks) with sessions held 2-3 times per week.

Monitor participants’ adherence to the intervention protocol and document any adverse effects or issues encountered.

6. Outcome Measures

- Reassess foot function and overall well-being at the end of the intervention period using the FFI and SF36 questionnaires.
- Compare post-intervention results with baseline measurements to evaluate the effectiveness of dynamic taping and TheraBand training.

7. Data Analysis

- Analyze demographic data and outcomes using descriptive statistics.
- Use Pearson Correlation tests to identify associations between risk factors and changes in foot function and quality of life.

8. Reporting and Dissemination

- Compile findings into a comprehensive report detailing the impact of dynamic taping and TheraBand training on CHP.
- Share results with participants, relevant stakeholders, and publish findings in appropriate academic forums.

**Study Protocol**

Ethical approval will be obtained from the Departmental Ethics Committee, Galgotias University

An informed consent will be signed by each participant and complete procedure of the study will be explained to the participants

Demographic data will be obtained from young NCC Cadets


Primary outcome measure Foot Function Index (FFI) will be used to evaluate disability, pain, activity restriction associated with chronic heel pain


Other outcome measures like SF36 will be used to evaluate quality of life

Results will be analyzed using estimate of prevalence in %ages, SD mean of the variables will be calculated. Student’s t- test will be used for pre and post analysis of the outcomes. Correlation test will be used to analyze associated risk factors

Compile findings into a comprehensive report detailing the impact of dynamic taping and TheraBand training on CHP.

**Research Implications:** This pilot study intends to provide an over view of enhancing the quality of life among young NCC Cadets by improving the disability associated with chronic heel pain and pave the way for conducting a full randomized controlled trail.

**Conflict of Interest:** Authors declares that there is no conflict of interest**.**

**Funding:** Funding will be obtained from Princess Nourah bint Abdulrahman University .

**References**

1. Balius R, Bossy M, Pedret C, Porcar C, Valle X, Corominas H. Heel fat pad syndrome beyond acute plantar fascitis. Foot (Edinb). 2021 Sep;48:101829.

2. Latt LD, Jaffe DE, Tang Y, Taljanovic MS. Evaluation and Treatment of Chronic Plantar Fasciitis. Foot ankle Orthop. 2020 Jan;5(1):2473011419896763.

3. Nakhaee M, Mohseni-Bandpei M, Mousavi ME, Shakourirad A, Safari R, Kashani RV, et al. The effects of a custom foot orthosis on dynamic plantar pressure in patients with chronic plantar fasciitis: A randomized controlled trial. Prosthet Orthot Int. 2023 Jun;47(3):241–52.

4. Jayaseelan DJ, Fernandez-de-Las-Penas C, Blattenberger T, Bonneau D. Thompson JV, Saini SS, Reb CW, Daniel JN. Diagnosis and management of plantar fasciitis. J Am Osteopath Assoc. 2014 Dec;114(12):900-6. doi: 10.7556/jaoa.2014.177. PMID: 25429080. J Sport Rehabil. 2021 Feb;30(5):812–7.

5. Thompson J V, Saini SS, Reb CW, Daniel JN. Diagnosis and management of plantar fasciitis. J Am Osteopath Assoc. 2014 Dec;114(12):900–6.

6. Lim AT, How CH, Tan B. Management of plantar fasciitis in the outpatient setting. Singapore Med J. 2016 Apr;57(4):168–70; quiz 171.

7. Tu P, Bytomski JR. Diagnosis of heel pain. Am Fam Physician. 2011 Oct;84(8):909–16.

8. Anselmo DS, Thatcher L, Erfle D. Gastrocnemius Recession as an Alternative to Midfoot Arthrodesis for Painful Midfoot Arthritis. J Foot Ankle Surg. 2020;59(5):1106–8.

9. McClinton S, Weber CF, Heiderscheit B. Low back pain and disability in individuals with plantar heel pain. Foot [Internet]. 2018;34:18–22. Available from: https://doi.org/10.1016/j.foot.2017.09.003

10. Walsh TP, Arnold JB, Gill TK, Evans AM, Yaxley A, Hill CL, et al. D. B. Irving, J. L. Cook, M. A. Young, and H. B. Menz, “Obesity and pronated foot type may increase the risk of chronic plantar heel pain : a matched case-control study,” vol. 8, pp. 1–8, 2007, doi: 10.1186/1471-2474-8-41. Rheumatol Int. 2017;37(7):1175–82.

11. Irving DB, Cook JL, Young MA, Menz HB. Obesity and pronated foot type may increase the risk of chronic plantar heel pain: A matched case-control study. BMC Musculoskelet Disord. 2007;8:1–8.

12. Hill CL, Gill TK, Menz HB, Taylor AW. Prevalence and correlates of foot pain in a population-based study: The North West Adelaide health study. J Foot Ankle Res. 1(1):1–7.

13. Abbafati C, Abbas KM, Abbasi M, Abbasifard M, Abbasi-Kangevari M, Abbastabar H, et al. Global burden of 369 diseases and injuries in 204 countries and territories, 1990–2019: a systematic analysis for the Global Burden of Disease Study 2019. Lancet. 2020;396(10258):1204–22.

14. Hossain M, Makwana N. “Not Plantar Fasciitis”: The differential diagnosis and management of heel pain syndrome. Orthop Trauma [Internet]. 2011;25(3):198–206. Available from: http://dx.doi.org/10.1016/j.mporth.2011.02.003

15. Gates LS, Arden NK, Hannan MT, Roddy E, Gill TK, Hill CL, et al. Prevalence of Foot Pain Across an International Consortium of Population-Based Cohorts. Arthritis Care Res. 2019;71(5):661–70.

16. Michelsson O, Konttinen YT, Paavolainen P, Santavirta S. Plantar heel pain and its 3-mode 4-stage treatment. Mod Rheumatol. 2005;15(5):307–14.

17. Kim DH, Lee Y. Effect of Dynamic Taping versus Kinesiology Taping on Pain, Foot Function, Balance, and Foot Pressure in 3 Groups of Plantar Fasciitis Patients: A Randomized Clinical Study. Med Sci Monit Int Med J Exp Clin Res. 2023 Nov;29:e941043.

18. Castro-Méndez A, Palomo-Toucedo IC, Pabón-Carrasco M, Ortiz-Romero M, Fernández-Seguín LM. The Short-Term Effect of Dynamic Tape versus the Low-Dye Taping Technique in Plantar Fasciitis: A Randomized Clinical Trial. Int J Environ Res Public Health. 2022 Dec;19(24).

19. Top E, Akil M. Effect of theraband exercises on muscle strength and motor development of individuals with moderate intellectual disability. Somatosens Mot Res. 2023 Mar;1–8.

20. Han SS, Her JJ, Kim YJ. [Effects of muscle strengthening exercises using a Thera Band on lower limb function of hemiplegic stroke patients]. Taehan Kanho Hakhoe Chi. 2007 Oct;37(6):844–54.
